# Supplementary figures and images for: Prognostic and therapeutic potential of gene profiles related to tertiary lymphoid structures in colorectal cancer
Source: PeerJ. 2024 Oct 31;12:e18401. doi: 10.7717/peerj.18401 (PMC11531753; doi:10.7717/peerj.18401)

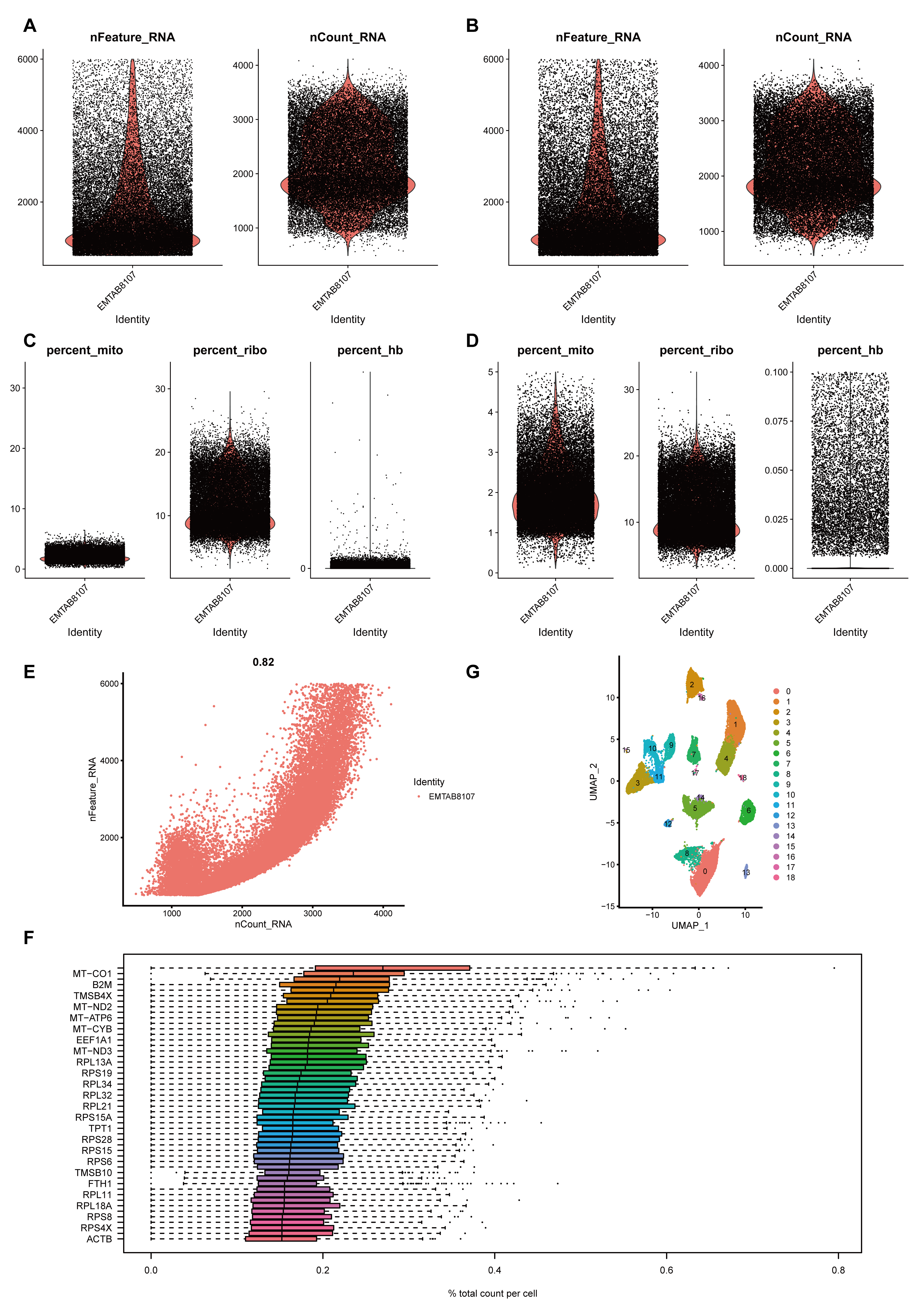

Supplement: Supplemental Information 1 — (A-B) nFeature_RNA and nCount_RNA of Single-cell data filtered according to the previously described filtering criteria. (A) Before filtering, (B) after filtering. (C-D) percent.mito, percent_ribo, and percent_hb of single-cell data filtered according to the preceding filtering criteria. (C) before filtering, (D) after filtering. The filtered data were used for subsequent analysis. (E) Correlation between nFeature_RNA and nCount_RNA, which is proportional to each other. (F) Demonstration of TOP highly expressed genes. [file peerj-12-18401-s001.png]

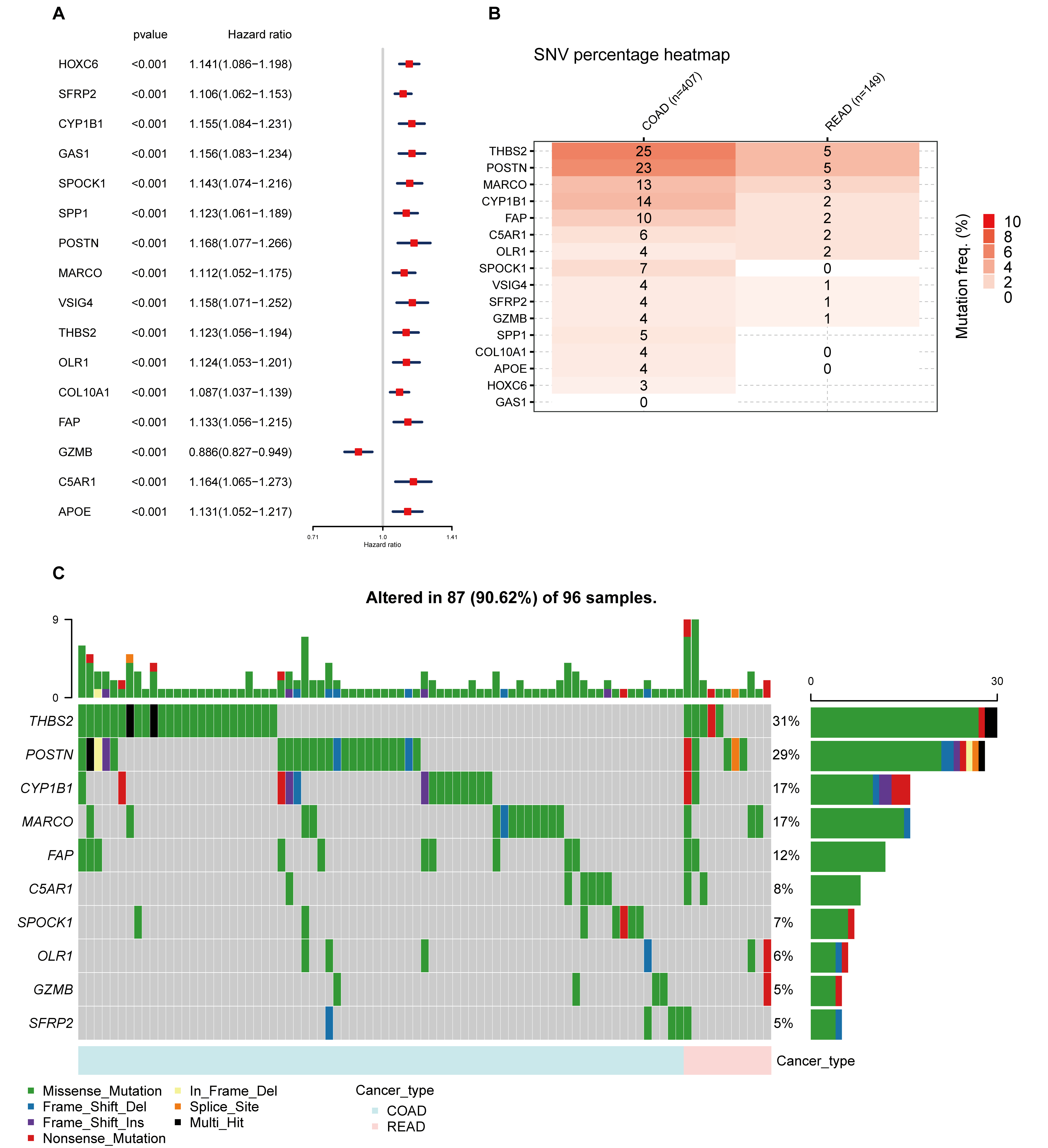

Supplement: Supplemental Information 2 — (A) Univariate cox regression of TLS-DEGs in colorectal cancer, forest plot showed the HRs of 16 TLS-DEGs screened by adjusted p value < 0.001. Abbreviations: HR, hazard ratio. (B) The profile of SNV of the TLS-DEGs in COADREAD. (C) Oncoplot presented the mutation distribution of the TLS-DEGs. [file peerj-12-18401-s002.png]

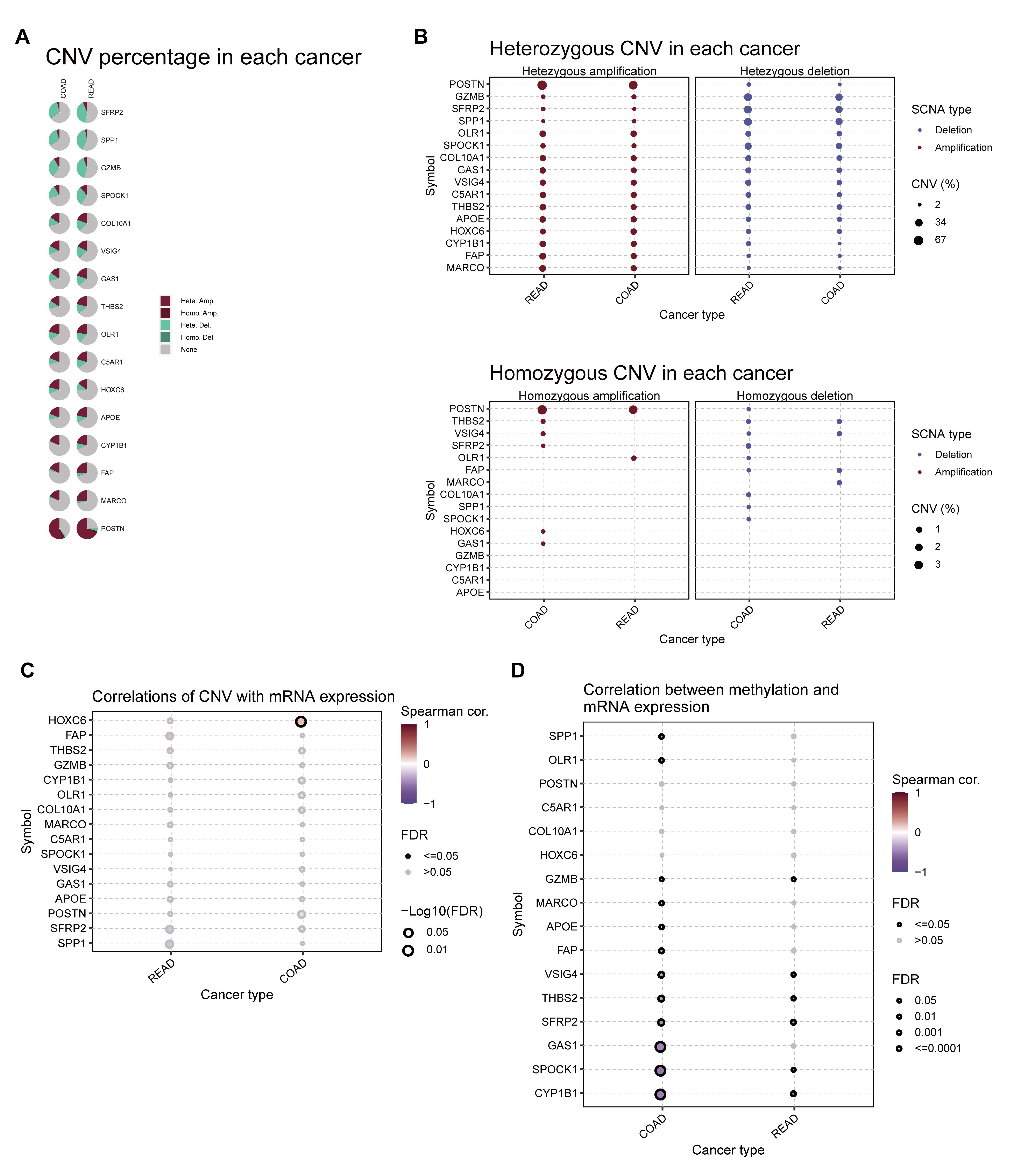

Supplement: Supplemental Information 3 — (A) CNA percentage of prognostic TLS-DEGs in COADREAD, the red color represented the ratio of copy number amplification. (B) Heterozygous (up) and homozygous (down) CNV of TLS-DEGs in COADREAD. The correlation between CNV (C), methylation (D) with mRNA expression each TLS-DEGs. The size of the circle indicates the P-value and the color represents the correlation coefficient. [file peerj-12-18401-s003.png]

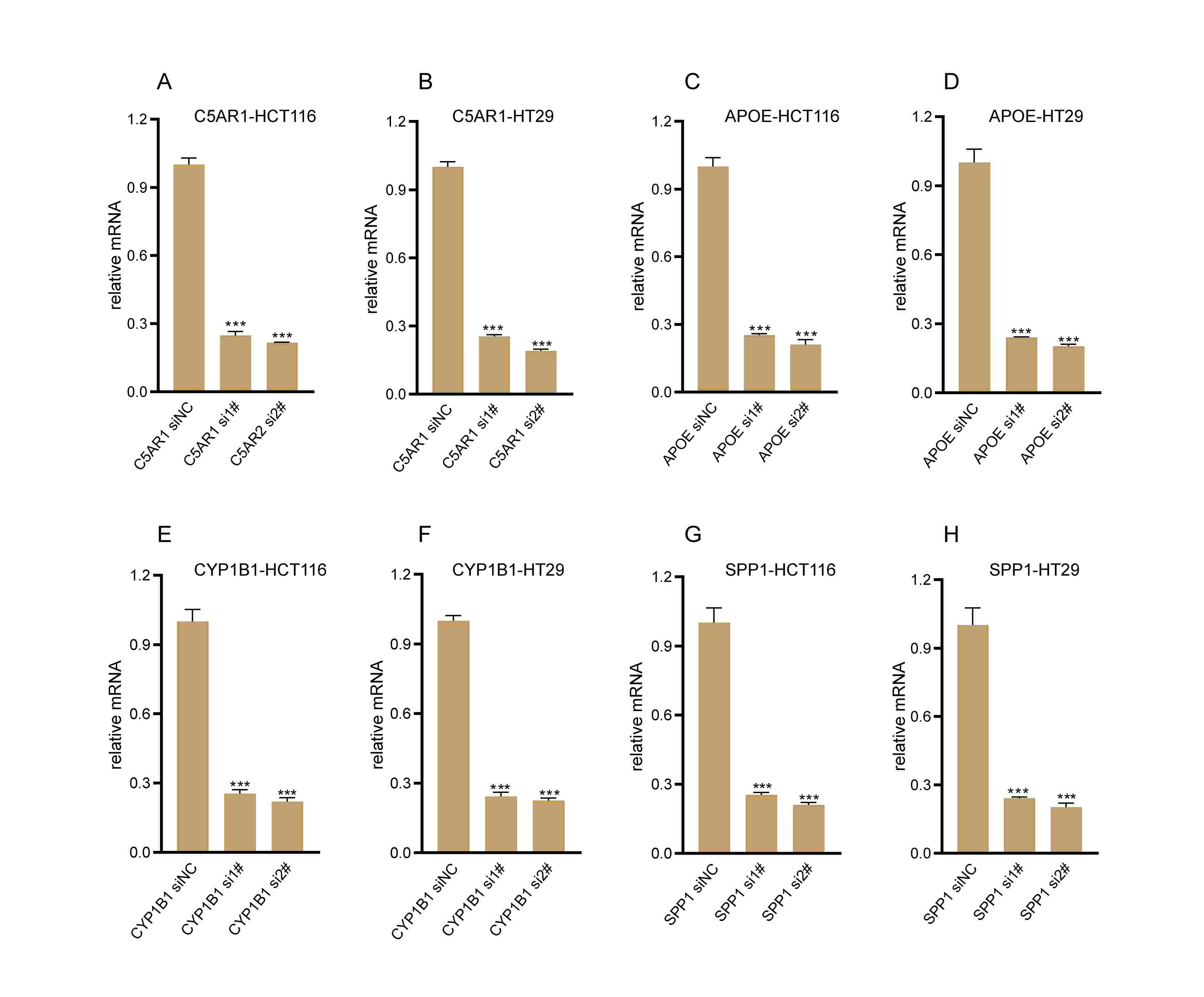

Supplement: Supplemental Information 4 — qRT-PCR to evaluate the mRNA level of C5AR1 (A, B), APOE (C, D), CYP1B1 (E, F), SPP1 (G, H) after transfection in HCT116 cells and HT29 cells separately. All siRNA sequences could result in significant decrease in the mRNA expression above (P<0.001). [file peerj-12-18401-s004.png]
