# Supplementary material for: Prognostic and therapeutic potential of gene profiles related to tertiary lymphoid structures in colorectal cancer
Source: PeerJ. 2024 Oct 31;12:e18401. doi: 10.7717/peerj.18401 (PMC11531753; doi:10.7717/peerj.18401)
Supplement: Supplemental Information 7 [file peerj-12-18401-s007.docx]

Supplementary material

q-RT PCR primers and siRNA sequences

| Gene | Sense | Antisense |
| --- | --- | --- |
| APOE | GTTGCTGGTCACATTCCTGG | GCAGGTAATCCCAAAAGCGAC |
| C5AR1 | TCCTTCAATTATACCACCCCTGA | ACGCAGCGTGTTAGAAGTTTTAT |
| CYP1B1 | TGAGTGCCGTGTGTTTCGG | GTTGCTGAAGTTGCGGTTGAG |
| SPP1 | CTCCATTGACTCGAACGACTC | CAGGTCTGCGAAACTTCTTAGAT |
| CD274 | TGGCATTTGCTGAACGCATTT | TGCAGCCAGGTCTAATTGTTTT |
| Gene | siRNA 1# | siRNA 2# |
| APOE | GUCACAUUCCUGGCAGGAUTT | GAUUACCUGCGCUGGGUGCUU |
| C5AR1 | GCCCATCTTGTTCACGTCCATTGTA | CCGACCGCTTTCTGCTGGTGTTTAA |
| CYP1B1 | CCCACAGCATGATGCGCAACTTCTT | CACAGCATGATGCGCAACTTCTTCA |
| SPP1 | CAGCCGTGGGAAGGACAGTTATGAA | CAGATTATATAAGCGGAAAGCCAAT |
